# Interinstitutional Variation of Caesarean Delivery Rates According to Indications in Selected Obstetric Populations: A Prospective Multicenter Study

**DOI:** 10.1155/2013/786563

**Published:** 2013-06-25

**Authors:** Gianpaolo Maso, Monica Piccoli, Marcella Montico, Lorenzo Monasta, Luca Ronfani, Sara Parolin, Carmine Gigli, Daniele Domini, Claudio Fiscella, Sara Casarsa, Carlo Zompicchiatti, Michela De Agostini, Attilio D'Atri, Raffaela Mugittu, Santo La Valle, Cristina Di Leonardo, Valter Adamo, Mara Fracas, Giovanni Del Frate, Monica Olivuzzi, Silvio Giove, Maria Parente, Daniele Bassini, Simona Melazzini, Secondo Guaschino, Caterina Businelli, Franco G. Toffoletti, Diego Marchesoni, Alberto Rossi, Sergio Demarini, Laura Travan, Giorgio Simon, Sandro Zicari, Giorgio Tamburlini, Salvatore Alberico

**Affiliations:** ^1^Multicenter Study Group on Mode of Delivery in Friuli Venezia Giulia, Institute for Maternal and Child Health (IRCSS) Burlo Garofolo, 34137 Trieste, Italy; ^2^Department of Obstetrics and Gynecology, Institute for Maternal and Child Health (IRCCS) Burlo Garofolo, 34137 Trieste, Italy; ^3^Epidemiology and Biostatistics Unit, Institute for Maternal and Child Health (IRCCS) Burlo Garofolo, 34137 Trieste, Italy; ^4^Department of Obstetrics and Gynecology, Hospital of Gorizia, 34170 Gorizia, Italy; ^5^Department of Obstetrics and Gynecology, Hospital of Latisana, 33053 Latisana, Italy; ^6^Department of Obstetrics and Gynecology, Hospital of Palmanova, 33100 Palmanova, Italy; ^7^Department of Obstetrics and Gynecology, Hospital of Monfalcone, 34070 Monfalcone, Italy; ^8^Department of Obstetrics and Gynecology, S. Giorgio Hospital, 33170 Pordenone, Italy; ^9^Department of Obstetrics and Gynecology, S. Maria degli Angeli Hospital, 33170 Pordenone, Italy; ^10^Department of Obstetrics and Gynecology, Hospital of S. Daniele del Friuli, 33038 S. Daniele del Friuli, Italy; ^11^Department of Obstetrics and Gynecology, S. Maria dei Battuti Hospital, 33078 S. Vito al Tagliamento, Italy; ^12^Department of Obstetrics and Gynecology, S. Antonio Abate Hospital, 33028 Tolmezzo, Italy; ^13^Department of Obstetrics and Gynecology, S. Maria della Misericordia Hospital, University of Udine, 33100 Udine, Italy; ^14^Department of Neonatology and Neonatal Intensive Care, Institute for Maternal and Child Health (IRCCS) Burlo Garofolo, 34137 Trieste, Italy; ^15^Regional Health Agency of Friuli Venezia Giulia, 33100 Udine, Italy; ^16^Department of Statistics and Programming, University La Sapienza, 00185 Rome, Italy; ^17^Research Direction, Institute for Maternal and Child Health (IRCCS) Burlo Garofolo, 34137 Trieste, Italy

## Abstract

The aim of the study was to identify which groups of women contribute to interinstitutional variation of caesarean delivery (CD) rates and which are the reasons for this variation. In this regard, 15,726 deliveries from 11 regional centers were evaluated using the 10-group classification system. Standardized indications for CD in each group were used. Spearman's correlation coefficient was used to calculate (1) relationship between institutional CD rates and relative sizes/CD rates in each of the ten groups/centers; (2) correlation between institutional CD rates and indications for CD in each of the ten groups/centers. Overall CD rates correlated with both CD rates in spontaneous and induced labouring nulliparous women with a single cephalic pregnancy at term (*P* = 0.005). Variation of CD rates was also dependent on relative size and CD rates in multiparous women with previous CD, single cephalic pregnancy at term (*P* < 0.001). As for the indications, “cardiotocographic anomalies” and “failure to progress” in the group of nulliparous women in spontaneous labour and “one previous CD” in multiparous women previous CD correlated significantly with institutional CD rates (*P* = 0.021, *P* = 0.005, and *P* < 0.001, resp.). These results supported the conclusion that only selected indications in specific obstetric groups accounted for interinstitutional variation of CD rates.

## 1. Introduction

Caesarean delivery (CD) rate is increasing worldwide [1]. In many developed countries, attention is focused on strategies to reduce this trend, given the concern that higher caesarean section rates increase maternal risks, without reducing perinatal mortality [2]. 

At present, the CD rate in Italy is one of the highest in the world and represents a challenge for the National Public Health system. In 1980, the rate was 11% and since then it has increased, reaching 39% in 2008, with significant interregional variations, ranging from 24% to 62% [1, 3]. In order to implement effective measures to reduce this trend and to evaluate temporal or interinstitutional variations of CD rates, several classifications were considered.

A recent systematic review evaluated the advantages and deficiencies of these methodologies [4]. Given the assumption that the aim of an “ideal” classification is to identify the groups of women who contribute significantly to the overall CD rate and to identify the main factors leading to CD in specific populations, the authors concluded that the 10-group classification (TGCS) [5], a women-based method, represents, at present, the best system for categorizing the mode of delivery, fulfilling the criteria of standardized, reliable, consistent, and action-oriented classification. Moreover, it has been stressed that this method facilitates auditing, analysing, and comparing CD rates across different settings and helps creating and implementing effective strategies to optimize CD rates [6, 7].

From its introduction in 2001 [5, 6], many studies focused on CD rates using this classification, but information on indications for performing CD on each woman according to inter-institutional differences in specific obstetric populations has not been available [8–17]. 

The aim of our study, carried out on more than 15 thousand deliveries from all of the 11 obstetric departments of our region, was to assess whether inter-institutional variation of overall CD rates correlated with the size or CD rates in selected obstetric populations of each institution, defined by using the TGCS. Moreover, we hypothesized that specific indications for CD in defined obstetric groups might account for overall variation of institutional CD rates.

## 2. Material and Methods

An 18-month prospective study collected data on mode of delivery from all births of the 11 single-institutional obstetric cohorts of Friuli Venezia Giulia (range 369–1,810 deliveries/year/unit). Friuli Venezia Giulia is a region of northeastern Italy accounting roughly for 10,000 deliveries per year with one of the lowest overall regional CD rates in Italy (23.4% in 2010). The source institutions, referred as institutions A to M, are first-level departments serving low-risk pregnancies, except for centers I and M working for a mixed population with the availability of a neonatal intensive care unit (NICU, second referral units).

The units differed for number of deliveries/year as follows: units A, B, C, E, F, G, H, and L had less than 1,000 deliveries/year; center D accounted for 1,000–1,500 deliveries/year; 1,500–2,000 deliveries/year were assisted in institutions I and M.

To avoid potential information bias due to different definitions on collected data, we created a regional standardized computerized database with the collaboration of all centers. All centers approved and validated the data collection form. All obstetricians and midwives of all the centers were instructed to manage the database and to collect data. Information on institutional deliveries was prospectively collected at the time of delivery by obstetrician or midwife attending the delivery in each center. Collected data were systematically reviewed every month by the referent obstetrician of each center. Special attention was devoted to overall data completeness and accuracy. During the study period, two of the authors (GM and SA) organized periodical multicenter meetings to discuss the results and provide assistance. All women provided informed consent to include their records in the presentation of summary data for births.

The study was approved by the institutional review board of the coordinating center (Technical Scientific Committee (CTS), Institute for Maternal and Child Health (IRCCS Burlo Garofolo, Trieste, project 86/05) February 28, 2007) and access to the data was approved by all hospital trust administrations. According to the Italian law on privacy (Art. 20-21, DL 196/2003), data were anonymized at every institution where each patient was assigned a unique identifier. This identifier did not allow to trace the patient's identity and other sensitive data. 

The study population of each institution was evaluated using the TGCS (Table 1) with specific reference to the following: (1) overall CD rate, (2) relative size of each group within each institutional cohort, and (3) CD rate in each group within the obstetric population of each institution. The relative size of the groups was calculated by dividing the number of subjects in each group by the overall obstetric population and expressed as a percentage. CD rates in each of the ten groups were calculated dividing the number of caesarean deliveries by the number of women in each of the groups.

Groups 2 (induced labour or prelabour CD in singleton, cephalic presentation, at term, nulliparous women) and 4 (induced labour or prelabour CD in singleton, cephalic presentation, at term, multiparous women) were further divided into groups 2a and 2b, and 4a and 4b, according to whether they were induced or delivered by pre-labour CD. 

In each group, the indications for induction of labour and CD were reported (Tables 2 and 3). For cases in which more than one indication was present, the obstetrician was asked to report the main indication for CD. With specific reference to dystocia, failure to progress was defined as either the absence of progressive cervical dilatation or progressive fetal descent in the active phase of labour [18]. Failed induction was considered as the failure to achieve the active phase of labour after use of vaginal prostaglandins and/or amniotomy and infusion of oxytocin [18, 19]. Each center was provided with a clinical practice algorithm for the assessment of these conditions; however, the management of failure to progress or failed induction was recorded by following the guidelines of the individual unit. Cardiotocographic (CTG) anomalies were categorized as suspicious and/or pathological according to specific guidelines for the interpretation of electronic fetal heart rate monitoring in labour [18]. 

CD was also classified as elective or emergency and before or during labour. 

Information on maternal age, gestational age at delivery, neonatal birthweight, and perinatal mortality was also collected.

Difference in means between centers was analyzed using the ANOVA if data were normally distributed, or else with the nonparametric Kruskal-Wallis test. Post hoc analysis was carried out using Bonferroni's correction. Difference in proportion between centers were analyzed using Pearson's chi-squared test or Fisher exact test as appropriate, and the Bonferroni correction was applied in case of multiple testing. Spearman's correlation coefficient was used to study the correlations between institutional CD rates and relative size/CD rates in each of the ten groups and to verify whether specific indications for CD in selected groups correlated with variation of overall CD rates.

Categorical variables were presented as frequencies and percentages, or as percentages and 95% confidence intervals; continuous variables were presented as mean and standard deviations.

Statistical analysis was carried out with the STATA statistical package (version 9.0) [20], and *P* < 0.05 (or *P* < 0.05/number of comparisons in case of Bonferroni correction) was considered statistically significant. 

## 3. Results

A total of 3,791 caesarean deliveries were registered among 15,726 deliveries, giving an overall CD rate of 24.1%. CD rates differed significantly among institutions (range, 14.3–34.1%).  Table 4 showed the distribution of maternal age, gestational age at birth, and neonatal birth weight among centers. Perinatal mortality rates were highest in centers serving very high-risk cases (I and M).

Distribution of the relative sizes and CD rates in each of the ten groups among centers are described in Supplemental Tables 1 and 2 (supplementary material available online at http://dx.doi.org/10.1155/2013/786563).

Pairwise comparisons among centers showed that differences in the distribution of ten groups relative sizes existed. Nulliparous women at term, cephalic presentation, spontaneous or induced labour (groups 1 and 2a, resp.) and multiparous at term, cephalic presentation, spontaneous labour or with past CD (groups 3 and 5, resp.) were the most represented groups in all the centers (Supplemental Table 1). CD rates in groups 1, 2a, 4a, 5, 8, and 10 differed among institutions (Supplemental Table 2). 

As for the correlation between the relative size of the ten groups and the overall CD rates, only groups 2b (nulliparous women, single cephalic, ≥37 weeks, CD before labour; Spearman's rho 0.90, *P* < 0.001), 4b (multiparous women, single cephalic, ≥37 weeks, CD before labour; Spearman's rho 0.77, *P* = 0.006), and 5 (multiparous women with past scar, single cephalic, ≥37 weeks; Spearman's rho 0.89, *P* < 0.001) were significantly correlated with the overall CD rates.

**Figure 1:**
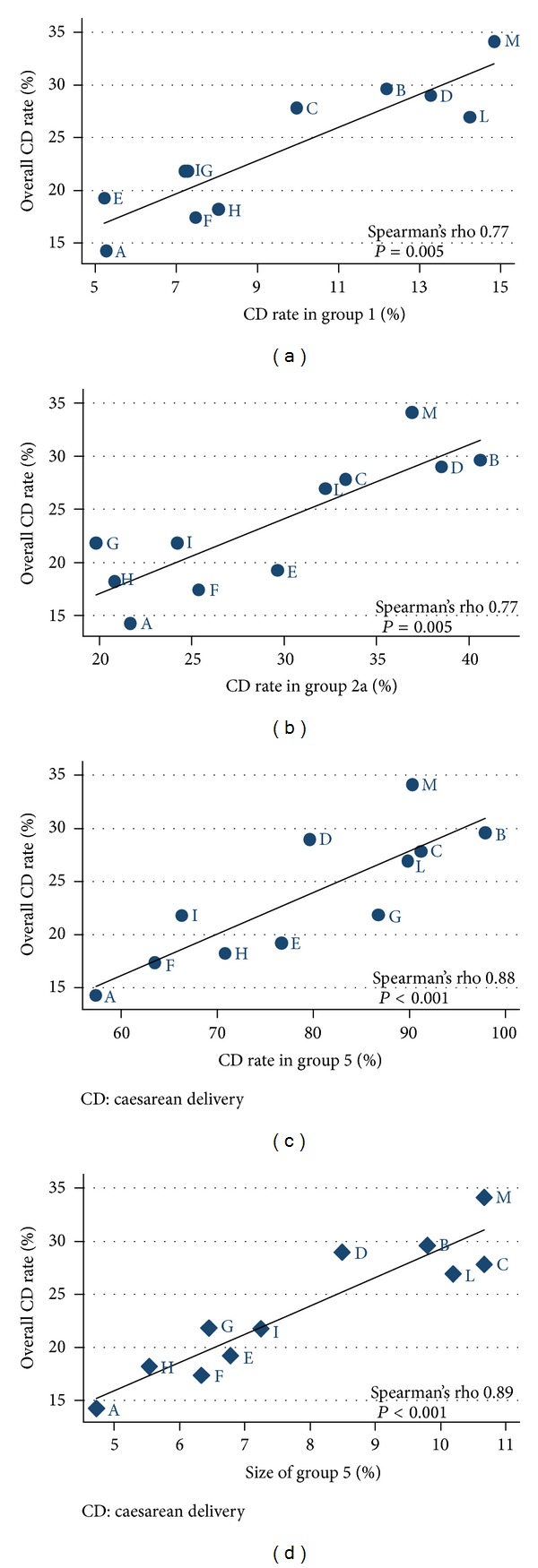
Correlation between overall inter-institutional CD rates and (a) CD rates in group 1 (nulliparous women at term, cephalic presentation, spontaneous labour), (b) CD rates in group 2a (nulliparous women at term, cephalic presentation, induced labour), and (c) CD rates and (d) relative size of group 5 (multiparous women at term, cephalic presentation, past CD). CD: caesarean delivery.

Overall CD rates correlated with CD rates in groups 1 (nulliparous women, single cephalic, ≥37 weeks, spontaneous labour; Spearman's rho 0.77; *P* = 0.005), 2a (nulliparous women, single cephalic, ≥37 weeks, induced labour; Spearman's rho 0.77; *P* = 0.005), and 5 (multiparous with past scar, single cephalic, ≥37 weeks; Spearman's rho 0.88; *P* < 0.001—Figure 1). 

Looking at the indications for CD and to their association with overall institutional CD rates, we decided to focus our attention on nulliparous women, single cephalic, ≥37 weeks, spontaneous or induced labour (groups 1-2a) and multiparous women with past scar, single cephalic, ≥37 weeks (group 5). Nulliparous and multiparous women at term, cephalic presentation, CD before labour (groups 2b and 4b, resp.) were excluded from further analysis, firstly because the sizes of these groups were not clinically relevant (supplemental Table 1, fourth column) and secondly because, in most cases, the indications for CD were unlikely to be susceptible to modification of management (i.e., elective or emergency prelabour CD for absolute fetal or maternal indications).

**Figure 2:**
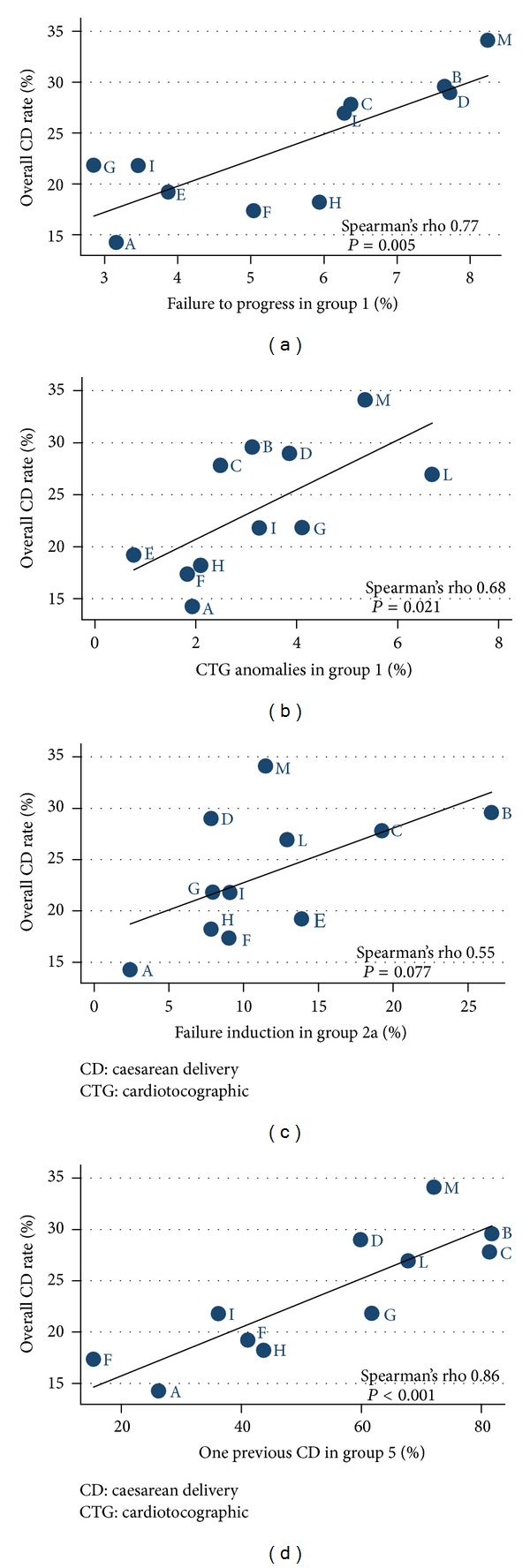
Correlation between indications in selected groups and overall inter-institutional CD rates. (a) Failure to progress and (b) CTG anomalies in group 1 (nulliparous women at term, cephalic presentation, spontaneous labour); (c) failed induction in group 2a (nulliparous women at term, cephalic presentation, induced labour); (d) one previous CD in group 5 (multiparous women at term, cephalic presentation, past caesarean delivery). CD: caesarean delivery; CTG: cardiotocographic.

Looking at the indications in group 1 (spontaneous labouring nulliparous women with a single cephalic pregnancy, at term), “failure to progress” and “CTG anomalies” were both significantly correlated with overall variation of CD rates (Spearman's rho 0.77; *P* = 0.005 and Spearman's rho 0.68; *P* = 0.021, respectively—Figures 2(a) and 2(b)).

The analysis of indications for CD in nulliparous women at term, in induced labour, showed that no specific indication correlated significantly with overall CD rates. Only “failed induction” demonstrated a correlation with overall CD rates, but it was not statistically significant probably due to the limited sample size of the group (Spearman's rho 0.55; *P* = 0.077—Figure 2(c)). Of interest was the observation that the rate of induction for “no absolute indications” in group 2a was 7.8% (112/1421). The CD rate in this cohort was 29.4.7% (33/112) and “failed induction” was the main indication for CD (19/33: 57.5%).

In the group of multiparous women, cephalic presentation at term, with at least one previous scar (group 5), there was a highly significant correlation found with the indication “one past CD” (correlation with overall CD rates: Spearman's rho 0.86; *P* < 0.001—Figure 2(d)). Moreover, inter-institutional variation of overall CD rates was significantly correlated with the proportion of CDs performed electively in group 5 for this indication alone (Spearman's rho 0.86; *P* < 0.001).

## 4. Comment

Our prospective evaluation of more than 15 thousand deliveries in a region with overall low CD rates offers new insight into the application of the TGCS. By assessing the mutually exclusive obstetric populations and providing an accurate registration of the main indications for CD, we were able to identify which groups contributed significantly to variation of overall CD rates and why overall CD rates differed among institutions. 

As suggested by the TGCS, we assessed firstly whether overall institutional CD rates were correlated with relative sizes or CD rates in specific groups. Overall CD rates correlated with CD rates in group 1 (nulliparous women, at term, single cephalic, spontaneous labour), in group 2a (nulliparous women, at term, single cephalic, induced labour), and with relative size and CD rates in group 5 (multiparous women, at term, cephalic presentation, with previous scar). These results strengthen the evidence that overall inter-institutional differences in CD rates depended on CD rates variations in these groups and correlated significantly with the size of multiparous women with past CD (group 5), a group at high risk for repeat CDs. Our observation confirmed that in order to reduce the overall CD rate, limiting CD rate in nulliparous women with a single cephalic pregnancy at term is the key to lowering the trend of overall increased abdominal deliveries. The decrease of CD rates in this group will consequently reduce the number of multiparous women with a previous CD (group 5) and hence the repeat CDs. These findings are in agreement with the results and conclusions of other studies. Brennan et al. and Delbaere et al. observed similar results, but they did not provide information about the causes of CD in these groups [15, 17]. Zhang et al. described the contemporary CD practice in the United States and assessed the indications of CD according to the TGCS, but their experience was based on an observational study, not taking into account the inter-institutional variation of CD rates [12].

After having identified the groups that contributed to the overall variation of inter-institutional CD rates, we focused our attention on indications leading to CD in these cohorts. The aim of this evaluation was to understand whether specific indications leading to CD in selected groups correlated with inter-institutional variation of CD rates. We realized the requirement for clear, unambiguous, and precise definitions for common obstetrical diagnoses and procedures. Standardization of these definitions was an essential step to prospectively collect reliable data and to allow a consistent comparison among institutions. This was necessary to overcome the potential bias generated by differences in definitions or coding and to improve inter-institutional reproducibility of information about the obstetric conditions leading to CD [21, 22].

In the group of nulliparous women, at term, single cephalic, spontaneous labour (group 1), the indications “CTG anomalies” and “dystocia-failure to progress” contributed significantly to the overall variation of institutional CD rates. This evidence might support the hypothesis that both the management of suspicious or pathological fetal heart rate tracings and of abnormal labour differed among centers. Barber et al. found similar results. They observed that among the primary caesarean deliveries, more subjective indications (“nonreassuring fetal status” and “arrest of dilation”) contributed significantly more than other more objective indications (malpresentation, maternal-fetal, and obstetric conditions) [23]. 

Another condition contributing to the inter-institutional variation of CD rates was the elective indication “one past CD” in the group of multiparous, at term with previous CD. A policy of elective CD in women with previous CD was significantly associated with the increase of overall CD rate. Clearly, the mode of delivery of women with past CD was planned differently among institutions. Some centers, with low elective CD rates for this indication, offered trial of labour, while others opted for elective CD, probably because they considered a failed trial of labour after previous CD was associated with more complications than elective repeat CD [24]. A recent study conducted in three Italian hospitals and dealing with the practitioners' attitudes toward the caesarean section showed that obstetricians would offer differently an elective CD to women with an uncomplicated single pregnancy in cephalic presentation, who had a previous CD, according to the indication of the primary CD [25].

It is well known that despite the presence of specific guidelines for managing anomalies of fetal heart patterns, abnormal labour, and women with a previous CD, inter-institutional variations in clinical evaluation and management of these conditions will exist [18, 19, 24, 26, 27]. These variations might be care giver dependent (i.e., dystocia: different thresholds to diagnose the arrest of dilation or fetal descent) or associated with other factors difficult to evaluate, such as hospital practices or organizational settings. It has been suggested that smaller institutions often lack resources required to respond to medical emergencies in the same manner as a tertiary care facility and would thus be more likely to recommend a CD with a lower medical threshold than a larger institution [28]. In our experience, no evidence supports this hypothesis. Different CD rates for specific indications in groups 1, 2a, and 5 were observed among centers with less than 1,000 deliveries per year as well as between institutions with more than 1,000 cases per year or NICU availability (centers I versus M). Unfortunately, neither data on application of guidelines for the management of dystocia and abnormal CTG in labour nor information on the decision making process leading to elective CD or trial of labour in multiparous women with scar were reported. However, the aim of our study was not to assess whether clinical management is appropriate or not, but to provide institutions with a tool to critically assess their obstetric practice in specific conditions. 

As for the group of nulliparous women at term, cephalic presentation, induced labour (group 2a), our data showed that induced labour in this cohort was at least three times as likely to result in CD than spontaneous labour (29.4% and 9.5%, resp.). However “failed induction” did not correlate significantly with overall CD rates probably because the contribution of this indication to the overall CD rate was only 5%. The evidence that the rate of CD for “failed induction” in this group differed among institutions might support the opinion that the decision to proceed with CD for this indication might be not based on uniform criteria and that clinical impatience may play a role in the decision making regarding the mode of delivery. Details on management of induction of labour were not available, but this finding might be useful to audit in each center marginal indications of induction, implementing an effective management to reduce unnecessary interventions [11, 12, 14].

The main limitation of our study was considering crude CD rates in each group without taking into account a number of variables that have been associated with high CD rates such as obesity, advanced maternal age, or clinical conditions defining the pregnancy as at risk [28–30]. However, the objective of our study was not to evaluate the adjusted CD rates of each institution, rather to provide a simple and immediate tool to assess which groups and indications contributed to the institutional CD rates [31]. Moreover, we demonstrated that the TGCS risk adjustment either with or without the inclusion of maternal characteristics and obstetrical risk factors, as predictors, might be considered as a reliable method to properly assess inter-institutional variation of CD rates [32].

Our results confirm that the TGCS represents a simple method that allows comparison of CD rates among institutions. The analysis of prospectively collected data, using standardized definitions of indications leading to CD and induction of labour in identified obstetric cohorts might be helpful to monitor and provide feedback to clinicians, identifying those procedures occurring without accepted medical indications [27, 31]. 

Our conclusions are in agreement with the remarks recently made by Robson et al. in their editorial and with the recommendations of the Society for Maternal Fetal Medicine and the American College of Obstetricians and Gynecologists. In this regard, every effort should be made to prevent the first CD in the nulliparous population by focusing the attention on caesarean deliveries occurring after labour inductions, those labeled as for “nonreassuring fetal status,” and those occurring for “labour arrest” or “failed induction” without meeting accepted criteria [31, 33]. This will translate in the reduction of primary CD rates with subsequent decrease of women with previous CD, at high risk of recurrence of CD and severe obstetric complication. In conclusion, our results support the concept that it is not relevant to argue about the rate of caesarean deliveries *per se*, but rather discuss how to reduce them by looking at the mode of delivery in a prospective process of labour ward audit [33, 34]. In this context, the assessment of the obstetric population according to the TGCS and the analysis of the indications leading to caesarean delivery allow to identify which groups of women contribute to the inter-institutional variation of CD rates and which are the reasons of this variation [35].

## Supplementary Material

Supplemental Table 1: Distribution of 10-groups relative sizes by center. Values are expressed as percentages (95% Confidence Intervals).
Supplemental Table 2: Caesarean delivery rate in the 10-groups by center. Values are expressed as percentages (95% Confidence Intervals). Group 9 is not considered because of low relative size.

## Figures and Tables

**Table 1:** 10-group classification. Groups 2 and 4 were further divided respectively in to 2a, 2b and 4a, 4b.

Group	Classification
1	Nulliparous, single cephalic, ≥37 weeks, in spontaneous labour
2a	Nulliparous, single cephalic, ≥37 weeks, induced labour
2b	Nulliparous, single cephalic, ≥37 weeks, CD before labour
3	Multiparous (excluding previous CD), single cephalic, ≥37 weeks, in spontaneous labour
4a	Multiparous (excluding previous CD), single cephalic, ≥37 weeks, induced labour
4b	Multiparous (excluding previous CD), single cephalic, ≥37 weeks, CD before labour
5	Previous CD, single cephalic, ≥37 weeks
6	All nulliparous breeches
7	All multiparous breeches (including previous CD)
8	All multiple pregnancies (including previous CD)
9	All transverse/oblique lies (including previous CD)
10	All preterm single cephalic, <37 weeks, including previous CD

CD: caesarean delivery.

**Table 2:** Indications of induction of labour.

(1) Prelabour rupture of membranes	
(2) Postterm (gestational age ≥ 41 weeks)	
(3) Hypertensive disorders	
(4) Other maternal reasons, for example, procedure done for the benefit of the mother*	
(5) Fetal reasons, for example, procedure done for the benefit of the fetus*	
(6) No absolute indications or no indication reported	

*Preexisting or gestational diabetes, preexisting maternal disease suggesting the termination of pregnancy, obstetric cholestasis, alloimmunisation, severe oligohydramnios, and intrauterine growth restriction.

**Table 3:** Indications of caesarean delivery.

(1) Suspicious or pathological cardiotocography (CTG anomalies) [18]	
(2) Other fetal reasons, for example, procedure done for the benefit of the fetus*	
(3) Other maternal reasons, for example, procedure done for the benefit of the mother*	
(4) Antepartal hemorrhage or placenta previa	
(5) Preeclampsia or HELLP syndrome	
(6) Breech presentation	
(7) One previous caesarean delivery	
(8) More than one caesarean delivery	
(9) Dystocia-failed induction [19]	
(10) Dystocia-failure to progress [18]	
(11) No indication reported including maternal request	

*HIV, preexisting or gestational diabetes, preexisting maternal disease suggesting the termination of pregnancy, obstetric cholestasis, alloimmunisation, severe oligohydramnios, and intrauterine growth restriction.

**Table 4:** Descriptive analysis of study population by center.

	Center											
	A	B	C	D	E	F	G	H	I	L	M	Overall
Deliveries, *n*	1291	950	957	1791	1328	1642	1054	868	2745	579	2521	15726
MA, yrs*	31.8 ± 5.0	31.7 ± 5.4	31.2 ± 5.3	31.7 ± 5.4	31.3 ± 5.1	31.8 ± 5.0	31.1 ± 5.2	31.6 ± 5.2	32.3 ± 5.1	31.8 ± 5.1	31.8 ± 5.4	31.7 ± 5.2
GA, wks ± days	39 ± 1	39 ± 1	39 ± 1	38 ± 6	39 ± 2	39 ± 1	39 ± 0	39 ± 2	39 ± 0	39 ± 0	38 ± 4	39 ± 0
GA, wks-range**	26–42	32–42	33–42	26–42	34–42	29–42	24–42	28–42	24–42	33–42	21–42	21–42
BW, grams*	3324 ± 469	3276 ± 452	3337 ± 451	3277 ± 531	3362 ± 447	3325 ± 481	3321 ± 460	3315 ± 493	3301 ± 560	3310 ± 443	3256 ± 631	3304 ± 520
PMR, *n* (%)^†^	1 (0.1)	0 (0)	1 (0.1)	8 (0.5)	3 (0.2)	4 (0.3)	2 (0.2)	1 (0.1)	15 (0.6)	0 (0)	16 (0.6)	51 (0.3)
CD rate, *n* (%)	184 (14.3)	281 (29.6)	266 (27.8)	519 (29.0)	255 (19.2)	285 (17.4)	230 (21.8)	158 (18.2)	598 (21.8)	156 (26.9)	859 (34.1)	3791 (24.1)
CD, 95% CI^§^	12.4–16.3	26.7–32.6	25.0–30.8	26.9–31.1	17.1–21.4	15.6–19.3	19.4–24.4	15.7–20.9	20.3–23.4	23.4–30.8	32.2–36.0	23.4–24.8

*ANOVA's *P* < 0.01 for all the variables; **Kruskal-Wallis's *P* < 0.01; ^§^Chi-squared *P* < 0.01; ^†^Fisher exact test *P* < 0.01.

MA: maternal age; GA: gestational age at delivery; Wks: weeks; BW: birthweight; PMR: perinatal mortality rate; CD: caesarean delivery; CI: confidence intervals. Maternal age, gestational age, and birthweight are expressed as mean ± standard deviation.
