# Supplementary material for: Interinstitutional Variation of Caesarean Delivery Rates According to Indications in Selected Obstetric Populations: A Prospective Multicenter Study
Source: Biomed Res Int. 2013 Jun 25;2013:786563. doi: 10.1155/2013/786563 (PMC3707216; doi:10.1155/2013/786563)
Supplement: Supplementary file 1 — Supplemental Table 1: Distribution of 10-groups relative sizes by center. Values are expressed as percentages (95% Confidence Intervals). Supplemental Table 2: Caesarean delivery rate in the 10-groups by center. Values are expressed as percentages (95% Confidence Intervals). Group 9 is not considered because of low relative size. [file 786563.f1.doc]

SUPPLEMENTAL TABLES

Table S1. Distribution of 10-groups relative sizes by center. Values are expressed as percentages (95 % Confidence Intervals).

| **10 Groups** | **Center** | | | | | | | | | | | |
| --- | --- | --- | --- | --- | --- | --- | --- | --- | --- | --- | --- | --- |
|  | **A** | **B** | **C** | **D** | **E** | **F** | **G** | **H** | **I** | **L** | **M** | **Overall** |
| **1** | 44.1  (41.3-46.8) | 37.2  (34.1-40.3) | 37.7  (34.6-40.9) | 36.2  (34.0-38.5) | 38.9  (36.3-41.6) | 39.9  (37.5-42.3) | 30.0  (27.2-32.8) | 32.9  (29.8-36.2) | 36.9  (35.1-38.7) | 41.3  (37.2-45.4) | 28.9  (27.1-30.7) | 36.1  (35.3-36.9) |
| **2 a** | 6.4  (5.1-7.9) | 6.7  (5.2-8.5) | 8.1  (6.5-10.1) | 8.5  (7.3-9.9) | 8.1  (6.7-9.7) | 7.4  (6.2-8.8) | 11.9  (10.1-4.1) | 13.2  (11.1-5.7) | 9.6  (8.5-10.8) | 5.3  (3.7-7.5) | 11.1  (9.9-12.3) | 9.0  (8.6-9.5) |
| **2 b** | 0.8  (0.4-1.4) | 3.9  (2.8-5.3) | 2.5  (1.6-3.7) | 2.6  (1.9-3.4) | 1.5  (0.9-2.3) | 1.0  (0.6-1.6) | 2.7  (1.7-3.7) | 1.8  (1.1-3.0) | 2.0  (1.5-2.6) | 1.7  (0.8-3.15) | 3.0  (2.4-3.8) | 2.1  (1.9-2.4) |
| **3** | 31.1  (28.6-33.7) | 26.8  (24.0-29.8) | 26.7  (24.2-29.9) | 27.3  (25.5-29.4) | 32.1  (29.6-34.7) | 31.6  (29.4-33.9) | 33.8  (30.9-36.7) | 31.2  (28.1-34.4) | 28.2  (26.5-29.9) | 31.3  (27.5-35.2) | 26.9  (25.2-28.7) | 29.3  (28.6-30.0) |
| **4 a** | 3.3  (2.4-4.4) | 4.5  (3.3-6.0) | 4.0  (2.8-5.4) | 3.6  (2.8-4.5) | 3.6  (2.7-4.8) | 4.0  (3.1-5.1) | 4.7  (3.5-6.2) | 5.8  (4.3-7.5) | 3.6  (2.9-4.3) | 1.4  (0.6-2.7) | 4.3  (3.6-5.2) | 3.9  (3.6-4.2) |
| **4 b** | 0.3  (0.1-0.8) | 1.4  (0.7-2.3) | 0.7  (0.3-1.5) | 0.7  (0.4-1.2) | 0.8  (0.4-1.5) | 0.5  (0.3-1.0) | 0.9  (0.4-1.6) | 0.6  (0.2-1.3) | 0.4  (0.2-0.8) | 0.7  (0.2-1.8) | 0.9  (0.5-1.3) | 0.7  (0.6-0.8) |
| **5** | 4.7  (3.6-6.0) | 9.8  (8.0-11.9) | 10.7  (8.8-12.8) | 8.5  (7.2-9.9) | 6.8  (5.5-8.3) | 6.3  (5.2-7.6) | 6.5  (5.0-8.1) | 5.5  (4.1-7.3) | 7.2  (6.3-8.3) | 10.2  (7.8-12.9) | 10.7  (9.5-11.9) | 7.9  (7.5-8.3) |
| **6** | 2.5  (1.7-3.5) | 3.8  (2.7-5.2) | 3.1  (2.1-4.4) | 2.6  (1.9-3.5) | 3.0  (2.2-4.1) | 2.7  (2.0-3.6) | 2.7  (1.8-3.8) | 3.1  (2.1-4.5) | 3.4  (2.8-4.2) | 1.0  (0.4-2.2) | 2.6  (2.0-3.3) | 2.9  (2.6-3.1) |
| **7** | 1.2  (0.7-2.0) | 0.6  (0.2-1.4) | 1.6  (0.9-2.6) | 1.1  (0.6-1.7) | 1.2  (0.7-1.9) | 1.2  (0.7-1.8) | 1.5  (0.9-2.5) | 1.2  (0.6-2.1) | 1.2  (0.8-1.7) | 1.6  (0.7-2.9) | 1.6  (1.2-2.2) | 1.3  (1.1-1.5) |
| **8** | 1.0  (0.5-1.7) | 1.2  (0.6-.19 | 0.9  (0.4-1.8) | 2.1  (1.5-2.8) | 0.8  (0.4-1.4) | 1.0  (0.6-1.7) | 0.7  (0.3-1.4) | 0.8  (0.3-1.7) | 1.9  (1.4-2.5) | 1.4  (0.6-2.7) | 2.6  (2.0-3.3) | 1.5  (1.3-1.7) |
| **9** | 0.0  (0.0-0.3) | 0.4  (0.1-1.1) | 0.0  (0.0-0.4) | 0.0  (0.0-0.2) | 0.0  (0.0-0.3) | 0.2  (0.0-0.5) | 0.0  (0.0-0.3) | 0.0  (0.0-0.4) | 0.2  (0.1-0.5) | 0.0  (0.0-0.6) | 0.0  (0.0-0.2) | 0.1  (0.05-0.1) |
| **10** | 4.6  (3.5-5.9) | 3.7  (2.6-5.1) | 3.7  (2.6-5.0) | 6.9  (5.7-8.1) | 3.2  (2.3-4.3) | 4.1  (3.2-5.2) | 4.8  (3.6-6.3) | 3.8  (2.6-5.3) | 5.3  (4.5-6.2) | 4.1  (2.7-6.1) | 7.5  (6.5-8.5) | 5.2  (4.9-5.6) |

Footnotes: *one sided 97.5% Confidence Intervals

Table S2. Caesarean delivery rate in the 10-groups by center. Values are expressed as percentages (95 % Confidence Intervals). Group 9 is not considered because of low relative size.

| **10 Groups** | **Center** | | | | | | | | | | | |
| --- | --- | --- | --- | --- | --- | --- | --- | --- | --- | --- | --- | --- |
|  | **A** | **B** | **C** | **D** | **E** | **F** | **G** | **H** | **I** | **L** | **M** | **Overall** |
| **1** | 5.3  (3.6-7.4) | 12.2  (9.0-16.1) | 10.0  (7.1-13.5) | 13.3  (10.8-16.1) | 5.2  (3.5-7.5) | 7.5  (5.6-9.8) | 7.3  (4.7-9.7) | 8.0  (5.2-11.8) | 7.2  (5.7-9.0) | 14.2  (10.1-19.3) | 14.8  (12.3-17.6) | 9.4  (8.6-10.1) |
| **2 a** | 21.7  (13.4-32.1) | 40.6  (28.5-53.6) | 33.3  (23.1-44.9) | 38.6  (30.8-46.8) | 29.6  (21.2-39.2) | 25.4  (18.0-34.1) | 19.8  (13.3-27.9) | 20.9  (13.9-29.4) | 24.2  (19.2-29.9) | 32.3  (16.7-51.4) | 36.9  (31.2-42.9) | 29.4  (27.0-31.8) |
| **2 b** | 100*  (69.2-100) | 100*  (90.5-100) | 100*  (85.8-100) | 100*  (92.3-100) | 100*  (83.2-100) | 100*  (79.4-100) | 100*  (87.2-100) | 100*  (79.4-100) | 100*  (93.5-100) | 100*  (69.2-100) | 100*  (95.3-100) | 100.0*  (98.9-100.0) |
| **3** | 1.5  (0.5-3.2) | 2.0  (0.6-4.5) | 2.3  (0.9-5.0) | 4.3  (2.7-6.5) | 2.6  (1.3-4.6) | 2.1  (1.1-3.8) | 3.4  (1.8-5.8) | 1.1  (0.2-3.2) | 2.5  (1.5-3.8) | 5.0  (2.3-9.2) | 2.5  (1.5-4.0) | 2.6  (2.2-3.1) |
| **4 a** | 4.8  (0.6-16.2) | 4.7  (0.6-15.8) | 18.4  (7.7-34.3) | 17.2  (8.9-28.7) | 6.3  (1.3-17.2) | 4.5  (0.9-12.7) | 22.0  (11.5-36.0) | 2.0  (0.1-10.6) | 7.1  (2.9-14.2) | 12.5  (0.3-52.7) | 14.7  (8.6-22.7) | 10.5  (8.1-13.2) |
| **4 b** | 100*  (39.8-100) | 100*  (75.3-100) | 100*  (59.0-100) | 100*  (75.3-100) | 100*  (71.5-100) | 100*  (66.4-100) | 100*  (66.4-100) | 100*  (47.8-100) | 100*  (73.5-100) | 100*  (39.8-100) | 100*  (84.6-100) | 100.0*  (96.6-100.0) |
| **5** | 57.4  (44.1-70.0) | 97.8  (92.4-99.7) | 91.2  (83.9-95.9) | 79.6  (72.3-85.7) | 76.7  (66.6-84.9) | 63.5  (53.4-72.7) | 86.8  (76.4-93.8) | 70.8  (55.9-83.0) | 66.3  (59.3-72.9) | 89.8  (79.2-96.2) | 90.3  (86.2-93.6) | 79.9  (77.6-82.1) |
| **6** | 100*  (89.1-100) | 100*  (90.3-100) | 100*  (88.4-100) | 97.9  (88.7-99.9) | 100*  (91.2-100) | 100*  (92.0-100) | 100*  (87.7-100) | 100*  (87.2-100) | 96.8  (91.0-99.3) | 100*  (54.1-100) | 100*  (94.5-100) | 99.1  (97.7-99.8) |
| **7** | 100*  (79.4-100) | 100*  (54.1-100) | 100*  (78.2-100) | 100*  (82.4-100) | 100*  (79.4-100) | 100*  (82.4-100) | 100*  (79.4-100) | 100*  (69.2-100) | 100*  (89.4-100) | 100*  (66.4-100) | 100*  (91.4-100) | 100.0*  (98.2-100.0) |
| **8** | 84.6  (54.6-98.1) | 63.6  (30.8-89.1) | 88.9  (51.8-99.7) | 89.2  (74.6-97.0) | 100*  (69.2-100) | 76.5  (50.1-93.2) | 100*  (59.0-100) | 71.4  (29.0-96.3) | 75.0  (61.1-86.0) | 100*  (63.1-100) | 98.5  (91.7-100) | 87.2  (82.2-91.2) |
| **10** | 33.9  (22.1-47.4) | 31.4  (16.9-49.3) | 40.0  (23.9-57.9) | 52.0  (42.8-61.1) | 38.1  (23.6-54.4) | 30.9  (20.2-43.3) | 25.5  (14.3-39.6) | 30.3  (15.6-48.7) | 45.9  (37.6-54.3) | 50.0  (29.1-70.9) | 54.8  (47.4-62.0) | 43.2  (39.7-46.6) |

Footnotes: *one sided 97.5% Confidence Intervals
